# Effect of Silver Nitrate and Sodium Fluoride with Tri-Calcium Phosphate on *Streptococcus mutans* and Demineralised Dentine

**DOI:** 10.3390/ijms19051288

**Published:** 2018-04-25

**Authors:** Ollie Yiru Yu, Irene Shuping Zhao, May Lei Mei, Edward Chin-Man Lo, Chun-Hung Chu

**Affiliations:** Faculty of Dentistry, The University of Hong Kong, Hong Kong, China; yuyiru@hku.hk (O.Y.Y.); zhao110@hku.hk (I.S.Z.); mei1123@hku.hk (M.L.M.); hrdplcm@hku.hk (E.C.-M.L.)

**Keywords:** dentine caries, silver nitrate, sodium fluoride, fTCP

## Abstract

This study investigated the effect of 25% silver nitrate (AgNO_3_) and 5% sodium fluoride (NaF) varnish with functionalized tri-calcium phosphate (fTCP) on a *Streptococcus mutans* (*S. mutans*) biofilm and dentine caries lesion. Demineralised dentine specimens were treated with 25% AgNO_3_ and 5% NaF + fTCP (Group 1), 25% AgNO_3_ and 5% NaF (Group 2), 25% AgNO_3_ (Group 3), or water (Group 4). The specimens were subjected to a *S. mutans* biofilm challenge after treatment. The biofilm was then studied via scanning electron microscopy (SEM), confocal laser scanning microscopy (CLSM), and colony forming units (CFU). The specimens were assessed by micro-computed tomography, X-ray diffraction (XRD), and Fourier transform infrared spectroscopy (FTIR). SEM and CLSM revealed less biofilm in Groups 1 to 3. The log_10_ CFU of Groups 1 to 4 were 4.5 ± 0.7, 4.4 ± 0.9, 4.4 ± 0.9, and 6.7 ± 1.0, respectively (Groups 1, 2, 3 < 4, *p* < 0.001). The lesion depths of Groups 1 to 4 were 212.6 ± 20.1 µm, 280.8 ± 51.6 µm, 402.5 ± 61.7 µm, and 497.4 ± 67.2 µm, respectively (Groups 1 < 2 < 3 < 4, *p* < 0.001). XRD demonstrated silver chloride formation in Groups 1, 2, and 3. FTIR found the amide I: HPO_4_^2−^ values of the four groups were 0.22 ± 0.05, 0.25 ± 0.05, 0.41 ± 0.12, and 0.64 ± 0.14, respectively (Groups 1, 2 < 3 < 4; *p* < 0.001). In conclusion, this study revealed that AgNO_3_ and NaF + fTCP reduced the damage of dentine caries by cariogenic biofilm.

## 1. Introduction

Dental caries is a biofilm-mediated disease on susceptible tooth surfaces resulting from cariogenic bacteria [1,2]. Because microbial factors are involved in dental caries, silver nitrate (AgNO_3_) was introduced for caries management. AgNO_3_ is an antimicrobial agent with dehydrating and sclerosing properties. Dentists used AgNO_3_ for the treatment of caries and indirect pulp capping because it could permeate and fill the affected dentine with the antibacterial silver particles [3]. Although AgNO_3_ exhibits a strong antibacterial action, it has no obvious effect on remineralisation. Thus, a topical application of AgNO_3_ solution followed by NaF varnish was proposed on the basis of the outstanding antibacterial effect of AgNO_3_ and the remineralising property of NaF [4]. Fluoride varnish containing 5% sodium fluoride (22,600 ppm F) is a common topical fluoride that dentists have used for caries management for decades. A literature review concluded that NaF varnish may arrest the white spot lesions of enamel caries [5]. The adhesive property of NaF varnish prolongs its contact time on the tooth surface [6], thus enhancing the uptake of fluoride and promoting remineralisation. The theoretical advantage of using AgNO_3_ solution together with NaF varnish is that the varnish may prevent the washing away or dilution of the applied AgNO_3_ with saliva.

An in vitro study was conducted to investigate the chemical and histological changes of artificial dentine carious lesions after the application of 25% AgNO_3_ and 5% NaF [7]. The results revealed that the adjunctive application of AgNO_3_ and NaF could arrest dentine caries and prevent dentine collagen degradation. Not only the presence of fluoride, but also the availability of calcium and phosphate ions, affect the remineralisation of the tooth. Xerostomia, a condition of reduced salivary flow, can affect the availability of calcium and phosphate ions in the oral environment, which is often associated with a change in the composition of saliva. To enhance the remineralising effect, researchers introduced the calcium phosphate system into NaF varnish. However, unfavourable reactions of calcium and fluoride can occur (precipitation of insoluble calcium fluoride) when fluoride is added to the calcium phosphate system. It is essential that the calcium component is isolated from the fluoride component. In addition, the calcium and the fluoride should interact constructively to facilitate remineralisation on the tooth surface. With the advances taking place in mechanochemistry, functionalized tricalcium phosphate (fTCP) was developed by fusing organic molecules, such as fumaric acid, with native β-tricalcium phosphate (β-TCP). β-TCP is a bioactive mineralising substance that is compatible with fluoride in water-based situations due to its limited solubility compared with other calcium salts and minerals. A barrier around the calcium ions can be created by functionalizing β-TCP with fumaric acid molecules. This fumaric-acid coating prevents the premature reaction of calcium and fluoride. The coating breaks when it is applied to the tooth surface. Calcium, phosphate, and fluoride ions become available to promote remineralisation through the tooth’s uptake of these ions [8].

Although a study examined the effect of AgNO_3_ and NaF on dentine and revealed its remineralising effect [7], a literature search on PubMed found that no study investigated its anti-microbial effects. In addition, no synergistic effects of AgNO_3_ and NaF with fTCP have ever been studied. Thus, the objective of this study was to observe the antibacterial and remineralising effect of AgNO_3_ and NaF with fTCP, AgNO_3_ and NaF, and AgNO_3_ alone on artificial dentine lesions.

## 2. Results

**Figure 1:**
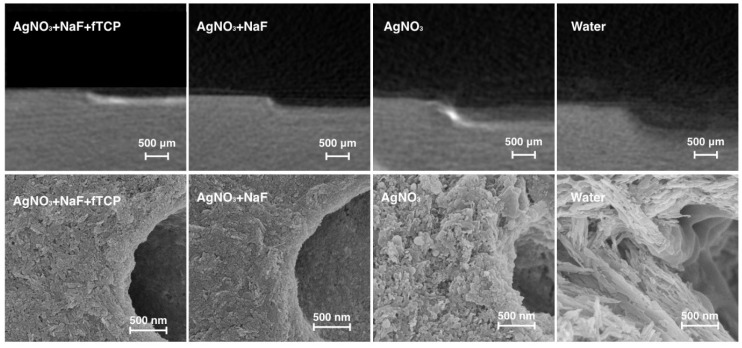
Representative micro-computed tomographs (upper row of the images) of artificial dentine carious lesion, and typical scanning electron micrographs (lower row of images) of surface morphology of artificial dentine caries of the four treatment groups.

The representative micro-computed tomography (micro-CT) images of the dentine blocks of the four groups are presented in Figure 1. Blocks in Group 1 revealed the lowest lesion depth among all the groups. The mean lesion depths (± standard deviation) of Group 1 to Group 4 were 212.6 ± 20.1 µm, 280.8 ± 51.6 µm, 402.5 ± 61.7 µm, and 497.4 ± 67.2 µm, respectively (*p* < 0.001; Groups 1 < 2 < 3 < 4).

The scanning electron microscopy (SEM) images revealed that the surfaces of the dentine blocks in Groups 1 and 2 were smooth and flat (Figure 1). Rough surfaces suggesting mineral loss were observed in bocks in Group 3. Collagens were found to be exposed and distributed in a disorderly fashion in blocks in Group 4, suggesting that the surfaces were substantially demineralised.

**Figure 2:**
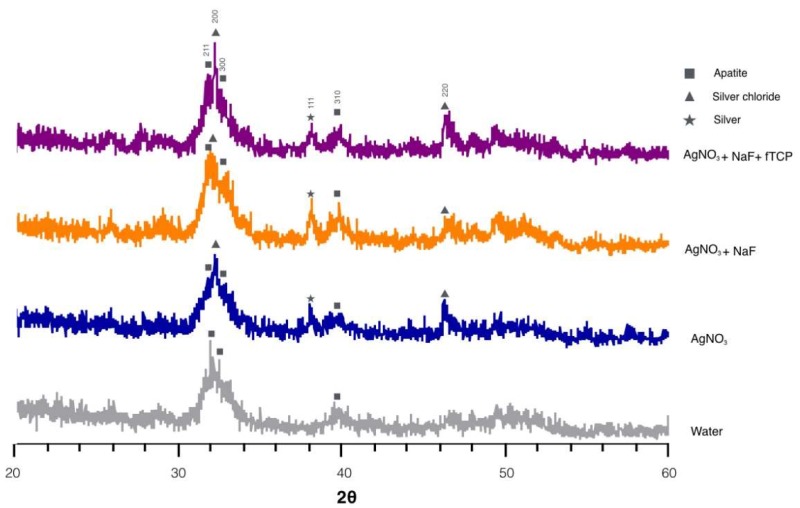
Typical X-ray diffraction patterns of the dentine in the four treatment groups.

Figure 2 reveals the typical X-ray diffraction (XRD) spectra of the four groups. The diffraction peaks were identified at 2θ = 25.8° (002), 2θ = 31.8° (211), 2θ = 32.2° (112), and 2θ = 32.8° (300), which corresponded to the peaks for hydroxyapatite (HA). The shape and intensity of peak 211, peak 112, and peak 300 in the spectra indicated that dentine in Groups 1 and 2 were better crystallized than that in Groups 3 and 4. In addition, prominent diffraction peaks were detected at 32.2° and 46.3° in the spectra of Groups 1, 2, and 3. These diffraction peaks corresponded to silver chloride (AgCl) (200) and (220) and indicated the presence of silver chloride. The peak at 2θ = 38.18° coincided with silver (111) in Groups 1, 2, and 3 and inferred the formation of metallic silver.

**Figure 3:**
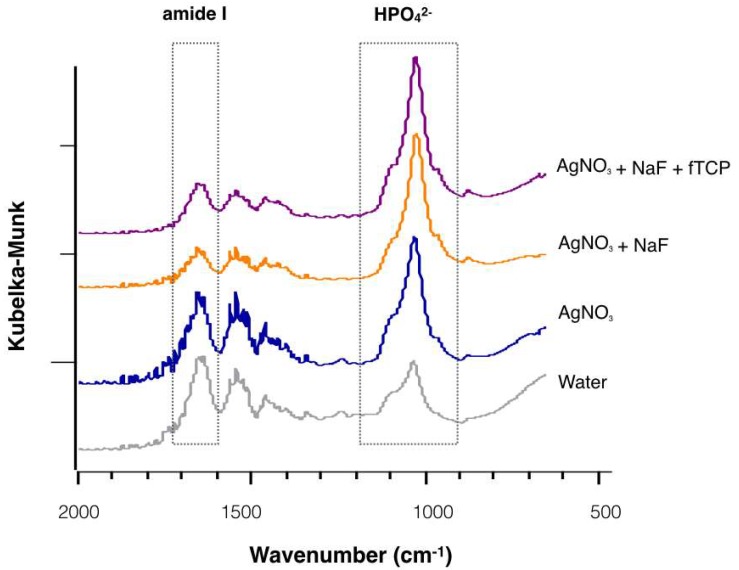
Typical Fourier transform infrared spectra of the artificial dentine caries of the four treatment groups. The peaks corresponding to the wavenumber 1585~1720 cm^−1^ represent amide I absorbance and the peaks between 900 and 1200 cm^−1^ represent HPO_4_^2−^ absorbance.

Figure 3 reveals the representative Fourier transform infrared spectroscopy (FTIR) spectra of the four treatment groups. The intensity of the phosphate band was strong, except in Group 4; the amide I band revealed a relatively higher peak in Group 3 and Group 4 than in Group 1 and Group 2. The mean values (± standard deviation) of amide I: HPO_4_^2−^ absorbance in Groups 1 to 4 were 0.22 ± 0.05, 0.25 ± 0.05, 0.41 ± 0.12, and 0.64 ± 0.14, respectively (*p* < 0.001; Groups 1, 2 < 3 < 4). The values of the ratio indicated that demineralised dentine in Group 4 had the largest amount of collagen degraded among the four groups.

**Figure 4:**
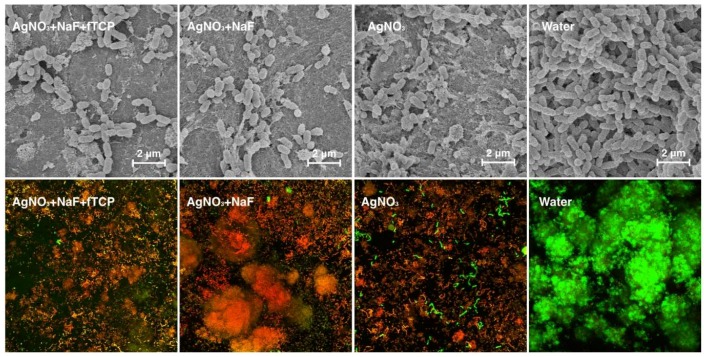
Representative scanning electron micrographs (images in the upper row) of the biofilm topography, and typical images of confocal laser scanning microscopy images (images in the lower row) of the *Streptococcus mutans* biofilm of the four treatment groups. Dead bacterial cells are marked red, and live cells are marked green (at magnification ×100).

The *Streptococcus mutans (S. mutans*) counts in the Log_10_ CFU (colony forming units) of the four treatment groups after seven days are displayed in Table 1. The number of *S. mutans* counts in the Log_10_ CFU of Groups 1 to 3 (i.e., treated with AgNO_3_) was significantly less than that of Group 4. SEM revealed the confluent growth of the bacteria in Group 4 (Figure 4), which corroborated the findings. Three-dimensional confocal laser scanning microscopy (CLSM) images (Figure 4) revealed that the red bacterial cells dominated in Groups 1, 2, and 3, indicating that most of the bacterial cells were dead in these three groups. The red to green ratios, representing dead to live ratios, are displayed in Table 1.

## 3. Discussion

In the present study, we used autoclave to sterilize the dentine blocks before and after the application of fluoride agents. Dentine is composed of approximately 50% organic materials and water by volume [9]. The main composition of the organic matrix is type I collagen. There were concerns that the high temperature during autoclave might denature the collagen and further affect the results of the study. However, a previous study was performed to test the the physical properties of extracted teeth after autoclave. It showed that the hydroxyapatite/collagen structure was weakened after autoclave, but it did not chemically destroy any major degree of collagen. This was because the heat and pressure could affect the ionic bond between hydroxyapatite and collagen, but the molecular structure of the collagen remained relatively unaffected [10].

Previous studies were performed to investigate the remineralising effect of NaF varnish with fTCP on artificial enamel caries lesions [11] and the caries preventive effect of NaF plus fTCP on dentine and artificial dentine caries [12]. Another study reported the effects of the adjunctive application of AgNO_3_ and NaF on dentine [7]. These studies used pH cycling models. The advantages of pH cycling models include simplicity, cost and time efficiency, stability, and reproducibility of the experiment. However, pH cycling models simplify the complicated biofilm metabolism and reflect the oral environment to a chemical level other than the biological level [13]. They ignore the microbiological part in the caries formation process and thus cannot be used to study the antimicrobial effect of the experimental treatment. Because this study aimed to investigate the antibacterial and remineralising effects of AgNO_3_ and NaF with fTCP, pH cycling models could not meet the needs of the experiment purpose. A biofilm model using *S. mutans* was adopted to create artificial caries lesions in the present study. *S. mutans* is the most commonly used cariogenic bacteria for caries research. The advantages of using a mono-species biofilm caries model are the simplicity of biofilm construction and stability of the biofilm composition. The single species biofilm has no problem relating to the uncontrollable fluctuating locus-specific of the oral environment. In addition, the growth rate of the bacteria and the physiological properties of the biofilm can be accurately investigated [14]. However, the oral flora in the mouth is much more complex. The interactions of different species of bacteria, such as competition, cross-feeding, or succession of the dental biofilm colony, cannot be considered in the mono-species biofilm model.

Results of this study showed that the growth of *S. mutans* biofilm on the dentine surface was retarded with AgNO_3_, with or without the fluoride agents (i.e., NaF or NaF with fTCP). The antibacterial effect of AgNO_3_ is attributed to its silver ions. Silver ions can bind intracellularly and extracellularly binding with thiol (sulfhydryl) groups of bacterial proteins and enzymes [15]. Silver ions could be actively uptaken by the bacteria and interact with the intracellular structure, such as proteins and enzymes, to inactivate them. Silver ions could also bind with nuclear acids and prohibit DNA replication. Furthermore, silver ions could inhibit the growth of bacteria cells by depositing in the cell membranes and walls electrostatically [16].

Fluoride varnish (NaF or NaF with fTCP) was applied after AgNO_3_ in this study. The fluoride varnish contains a high concentration of fluoride (22,600 ppm) which could inhibit the growth of biofilm through the inhibition of cellular enzymes in glycolysis and the reduction of acid production [17]. It was conceivable that the addition of fluoride varnish could have a complementary antibacterial effect. However, the results of this study did not demonstrate a synergistic antibacterial effect of NaF varnish and AgNO3 solution against *S. mutans* biofilms.

The AgNO_3_ + NaF with fTCP group had the lowest lesion depth (i.e., less demineralisation) among the four experimental groups. fTCP precludes unproductive calcium-fluoride interactions and allows remineralisation to create an acid-resistant mineral. Similar results were found in in vitro studies investigating the remineralisation effect of NaF with fTCP on enamel. Three studies reported that NaF with fTCP is superior than NaF in remineralising enamel using a pH cycling model [18,19,20], but one study found NaF with or without fTCP had a similar remineralising effect on enamel [11]. Another study reported NaF with or without fTCP had a similar remineralising effect on dentine [12]. This study used a chemical model, whereas the present study used a biofilm model in which the demineralisation process overwhelmed the remineralisation process. Moreover, the variation of the acidity of biofilm model was not as large as that in the chemical model. The fTCP increased the fluoride uptake and provided additional calcium ions. This facilitated the remineralisation of the artificial dentine caries lesion and produced a significantly lower lesion depth than that treated with NaF varnish.

Apart from mineral substances which are mainly composed of hydroxyapatite, dentine has a substantial amount of organic matrix which is mainly composed of type I collagen. The HPO_4_^2−^ band is representative of the mineral, and the amide I band represents the collagen in the FTIR spectrum [21]. The ratio of amide I: HPO_4_^2−^ indicates the degree of dentine demineralisation. The results of the amide I: HPO_4_^2−^ ratio in this study revealed that AgNO_3_ inhibited collagen degradation of dentine by the *S. mutans* biofilm. The effect might relate to the binding ability of AgNO_3_ to the bacterial enzyme. The silver ions can bind with the active side chain of the enzyme. Thus, the catalytic function of the bacterial collagenase might be inhibited by the silver ions [7]. The results of this study showed that collagen degradation was less in dentine treated by the adjunctive application of AgNO_3_ than that treated by AgNO_3_ alone. This phenomenon could be attributed to the inhibition effects of NaF on matrix-bound metalloproteinases (MMPs) [22]. The MMPs bound to the dentine collagen could be exposed and activated after demineralisation, causing degradation of the collagen fibrils [23]. The process of collagen degradation was slowed down by the suppression of MMPs. However, it was worth noting that the addition of fTCP did not have a protective effect on dentine collagen.

Previous in vitro studies proved that 25% AgNO_3_ solution followed by 5% NaF varnish was effective at preventing dentine demineralisation and dentine collagen degradation in a pH cycling model [7]. In addition, the AgNO_3_ solution and fluoride varnish are available in most countries at low-cost [4]. Caries management using AgNO_3_ solution and fluoride varnish is non-invasive, painless, simple, and of low cost. This study provided additional information on the adjunctive application of AgNO_3_ and NaF in clinical application. The results suggested that AgNO_3_ and NaF with fTCP could produce successful clinical outcomes to arrest dentine caries.

## 4. Materials and Methods

### 4.1. Sample Preparation and Treatment

**Figure 5:**
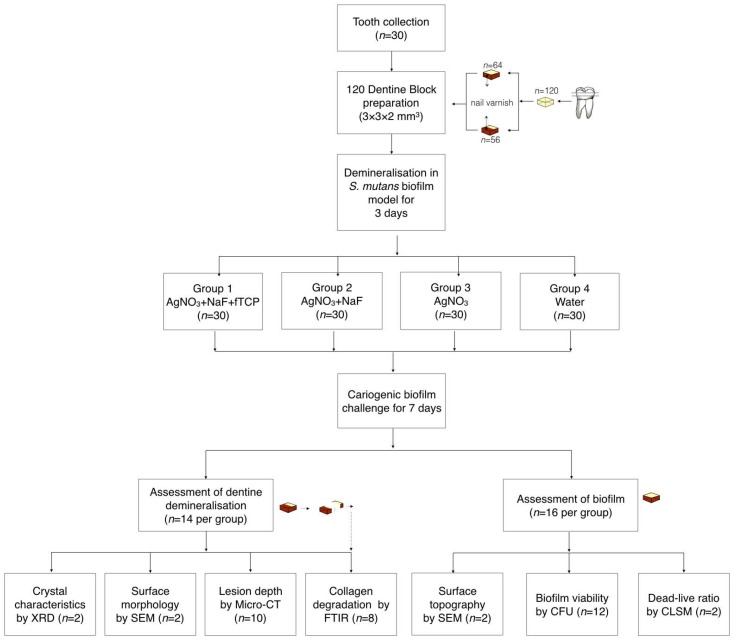
Flow chart of the study.

Dentine slices were prepared from extracted sound human third molars with ethics approval from the Institutional Review Board of the University of Hong Kong/Hospital Authority Hong Kong West Cluster (IRB number UW 17-089) from 11 Febuary 2017. Figure 5 is a flow diagram that summarizes the methods in this study.

The dentine slices (2 mm thick) were polished smooth, and each slice was sectioned into four dentine blocks (3 × 3 × 2 mm^3^). The set of four blocks from the same slice was excluded if cracks or other defects were found in the block(s) via a stereomicroscope (magnification ×10). A total of 120 dentine blocks were prepared, and 56 blocks were used for the assessment of dentine demineralisation. Half of the experimental surfaces of these 56 blocks were covered with an acid-resistant nail varnish (Clarins, Paris, France), and the varnished surfaces were used as the internal control. The remaining 64 dentine blocks were used for the assessment of the biofilm. *S. mutans* (American Type Culture Collection, ATCC 35668) was anaerobically cultured at 37 °C on blood agar plates for two days. A single colony was picked from each plate for a 24-hour broth culture in brain heart infusion (BHI) at 37 °C under the anaerobic condition. Subsequently, bacterial cell pellets were harvested and resuspended in BHI with 5% sucrose to a cell density of McFarland standard No. 2 (6 × 108 cells/mL). A 1-mL bacteria culture was inoculated on each dentine block sitting in a well of a 24-well plate. The plates were placed in an anaerobic chamber at 37 °C for three days to create a dentine lesion with a demineralisation zone of approximately 80 μm in depth [24]. The biofilm on the dentine surface was then removed via an autoclave and ultrasonication.

The four blocks obtained from the same dentine slide were randomly allocated into four groups for treatment. The blocks in Group 1 received a topical application of 25% AgNO_3_ (silver nitrate liquid; Gordon Laboratories, Upper Darby, PA, USA), which contains 151,130 ppm of silver followed by a 5% NaF varnish containing 22,600 ppm of fluoride with fTCP (Clinpro™ white varnish; 3M ESPE, St. Paul, MN, USA). The blocks in Group 2 received a topical application of 25% AgNO_3_ followed by 5% NaF varnish (Duraphat; Colgate-Palmolive Co., New York City, NY, USA) containing 22,600 ppm of fluoride. The blocks in Group 3 received a topical application of 25% AgNO_3_. The blocks in Group 4 received deionised water. All experimental solutions were applied onto the surface of blocks with a microbrush (Micro applicator–regular; Premium Plus International Ltd., Hong Kong, China). The experimental solutions were removed after 4 h. The blocks were then subjected to a cariogenic challenge using *S. mutans* biofilm as described in the sample preparation above for seven days. After the cariogenic challenge, the blocks were used for the assessment of dentine demineralisation or of the biofilm.

### 4.2. Assessment of Dentine Demineralization

The lesion depth, surface morphology, crystal characteristic, and dentine collagen degradation were studied during the assessment of dentine demineralisation.

#### 4.2.1. Lesion Depth

Ten blocks from each group were used to study the lesion depth via X-ray micro-CT (SkyScan 1172; SkyScan, Antwerp, Belgium). The voltage and current of the X-ray source were 80 kV and 100 uA, respectively. The image pixel size was set at 8 μm. The scanning results of the blocks were reconstructed using NRecon reconstruction software (SkyScan, Antwerp, Belgium). The reconstructed three-dimensional images were viewed and analysed with the CTAn data analysing software (SkyScan, Antwerp, Belgium). Fifteen images for each block were selected from the reconstructed cross-sectional images for the evaluation of the lesion depth. Image J (National Institutes of Health, Bethesda, MD, USA) was used to measure the lesion depth of the block. The internal control was used as a reference line.

#### 4.2.2. Surface Morphology

Two blocks from each group were fixed in 2.5% glutaraldehyde and were dehydrated using ethanol solutions (70% for 10 min, 85% for 10 min, 95% for 10 min, and 100% for 20 min). They were critical-point dried and sputter-coated with gold to study the surface morphology under SEM (Hitachi S-4800 FEG Scanning Electron Microscope; Hitachi Ltd., Tokyo, Japan).

#### 4.2.3. Crystal Characteristics

Two blocks from each group were used for XRD analysis to study the crystal characteristics. XRD data were collected with an X-ray powder diffractometer (D8 Advance; Bruker AXS, Karlsruhe, Germany) equipped with scintillation counting with a CuKa (l = 1.5418 Å) radiation detector. The accelerating voltage of the X-ray generator was 40 kV, and the applied current was 40 mA. The data were collected with 2θ range = 20–60°, step size = 0.02°, and scan speed = 30 s/step. The phase purity and indexing of the chemical phase was checked by using the International Center for Diffraction Data (ICDD, PDF-2 Release 2004) database (http://www.icdd.com/) match search. The XRD patterns were analyzed with the Bruker Diffrac plus EVA program (Bruker AXS, Karlsruhe, Germany) to study the crystal characteristics.

#### 4.2.4. Collagen Degradation

Eight blocks from each group were sectioned longitudinally, and the dentine collagen degradation was evaluated via FTIR spectroscopy (UMA 500, Bio-Rad Laboratories, Hercules, CA, USA) equipped with an attenuated total reflection element. The infrared radiation ranged from 650 to 4000 cm^−1^ in the wavelength number [25]. The spectra of the dentine carious lesions were obtained through the average acquisition of data at the spatial resolution achieved with a spot that was 32 μm in radius. The ratio of the integrated area of collagen amide I absorbance (between 1585 and 1720 cm^−1^) to that of HPO_4_^2−^ absorbance (between 900 and 1200 cm^−1^) was calculated. The value of the amide I: HPO_4_^2−^ absorbance ratio was used to evaluate the extent of the demineralisation of the dentine carious lesions [25].

### 4.3. Assessment of the Biofilm

The growth kinetics, surface topography, and viability of *S. mutans* on the dentine block were studied in the assessment of the biofilm.

#### 4.3.1. Growth Kinetics

Twelve blocks from each group were used to study growth kinetics of the biofilm by determining the colony-forming units (CFU) of the *S. mutans*. Serial 10-fold dilutions of homogenized biofilm samples in 1% sterile phosphate buffered solution were plated in duplicate with a spiral plater (Autoplate 4000; Spiral Biotech Inc., Norwood, MA, USA). Horse blood agar (Defib Horse Blood; Hemostat Laboratories, Dixon, CA, USA) plates were used, and the plates were cultivated anaerobically for 72 h before CFU counting.

#### 4.3.2. Surface Topography

Two blocks from each group were used to study the surface topography of the biofilm. The blocks with biofilm were fixed in 2.5% glutaraldehyde solution and dehydrated with ethanol solutions. The blocks were dried and sputter coated with gold for the study of the topographical features of the *S. mutans* biofilm under SEM.

#### 4.3.3. Viability of Bacteria

Two blocks from each group were used to study the viability of *S. mutans* in the biofilm using confocal laser scanning microscopy (CLSM) (Olympus, Tokyo, Japan). 

The biofilms were labelled in situ using two fluorescent probes: PI and SYTO-9 (LIVE/DEAD BacLight Bacterial viability kit; Molecular Probes, Eugene, OR, USA). The dead cells were labelled red with the PI probe, whereas the live cells were labelled green with the SYTO-9 probe [26]. After labelling, five cellular images of each biofilm specimen were obtained via CLSM and viewed via Fluoview FV 1000 (Olympus, Tokyo, Japan). The ratio of dead-to-live bacteria as indicated by the red-to-green ratio was examined using special image analysis software (Image J; National Institutes of Health, Bethesda, MD, USA). The ratio was used to study the antimicrobial effects of the experimental treatment.

### 4.4. Statistical Analyses

Computer software SPSS Statistics—V20.0 (IBM Corporation, Armonk, NY, USA) was used for the analyses. One-way analysis of variance with Bonferroni multiple comparison tests was used to analyze and compare the lesion depth value of the amide I: HPO_4_^2−^ absorbance ratio and the CFU counts across the four treatment groups. The level of statistical significance for all tests was set at 0.05.

## 5. Conclusions

AgNO_3_ and NaF with fTCP reduced the damage of dentine caries by cariogenic biofilm. The addition of NaF with fTCP did not affect the antibacterial effect of AgNO_3_.

## Figures and Tables

**Table 1:** Growth kinetics (log_10_ CFUs) and viability (dead-live ratio) of *S. mutans* in the biofilm of the four treatment groups after seven days.

Measurement	AgNO_3_ + NaF + fTCP	AgNO_3_ + NaF	AgNO_3_	Water	*p* Value
Log_10_ CFU	4.50 ± 0.73a	4.39 ± 0.93a	4.38 ± 0.92a	6.69 ± 0.97b	<0.001; a < b
Dead-live ratio	1.44 ± 0.62a	1.39 ± 0.73a	1.43 ± 0.25a	0.51 ± 0.04b	<0.001; a > b

## Abbreviations

AgNO_3_	silver nitrate
NaF	sodium fluoride
fTCP	functionalized tri-calcium phosphate
*S. mutans*	*Streptococcus mutans*
SEM	scanning electron microscopy
CLSM	confocal laser scanning microscopy
CFU	colony forming units
Micro-CT	micro-computer tomography
XRD	X-ray diffraction
FTIR	Fourier transform infrared spectroscopy
